# Synthesis and Characterization of Fly Ash-Based Geopolymers Activated with Spent Caustic

**DOI:** 10.3390/gels8090562

**Published:** 2022-09-02

**Authors:** Ruobing Zhang, Qian Wan, Yimin Zhang, Xuemian Zhang

**Affiliations:** 1 School of Resource and Environmental Engineering, Wuhan University of Science and Technology, Wuhan 430081, China; 2 State Environment Protection Key Laboratory of Mineral Metallurgical Resources Utilization and Pollution Control, Wuhan University of Science and Technology, Wuhan 430081, China; 3 Collaborative Innovation Center of Strategic Vanadium Resources Utilization, Wuhan 430081, China; 4 Hubei Provincial Engineering Technology Research Center of High Efficient Cleaning Utilization for Shale Vanadium Resource, Wuhan 430081, China

**Keywords:** spent caustic, alkali activator, geopolymer, immobilization, organics

## Abstract

The spent caustic with strong alkali first replaced the alkali activator to prepare the geopolymer. The influence of spent caustic to the geopolymer was characterized through compressive strength measurement, XRD, MIP analysis and NMR, and the immobilization efficiency of organic in geopolymer was evaluated through the measurement of total organic carbon (TOC). The results show that the spent caustic can partially replace the alkali activator to prepare the geopolymer, and it shows a better performance than that which was activated with pure NaOH solution when the alkalinity is between 4 mol and 14 mol. The organic matter in the spent alkali can be effectively fixed in the geopolymer, which will hinder the geopolymerization in the initial stage of the polymerization reaction but has little effect on the chemical structure and mechanical properties of the final product. With the degree of alkalinity increasing, the immobilization efficiency is improved, and the maximum can reach 84.5%. The organics in the spent caustic will hinder geopolymerization at the initial stage but has little effect on the chemical structure and mechanical property of the final product. This study proposes a new method for the recycling of spent caustic, which also reduces the preparation cost of geopolymers.

## 1. Introduction

Spent caustic is a kind of the liquid wastes generated in various chemical industries, especially in oil and gas refineries [1]. It may account for more than 90% of the total contamination generated by refineries in terms of oil and grease. Due to being used to remove organic sulfides, hydrogen sulfide and carbon dioxide, the typical spent caustic usually contains high alkalinity (5–12 wt% NaOH), sulfide content (0.5–4 wt% sulfide-S), phenols, oils and other organic contamination [2,3]. It has a high alkali (pH > 12) and chemical oxygen demand (COD), so it is classified as a hazardous waste according to the National Hazardous Waste List [4,5]. The technology for the treatment of spent caustic includes neutralization, incineration, Fenton oxidation and wet air oxidation (WAO). At present, wet air oxidation is widely used as a common treatment method for spent caustic [6,7]. After treatment by the wet air oxidation process, the sulfide is oxidized to sulfate so as to remove the odor, and the organics are also partially decomposed into CO_2_ and H_2_O to reduce COD. However, treated spent caustic still needs additional treatment, such as acid neutralition, before being discharged to the sewage treatment plant, due to its high COD level and high alkalinity [4]. This treatment process not only requires additional acid, and also does not utilize the high alkalinity of spent caustic, thereby causing waste of resources.

Geopolymers are a kind of environmentally friendly aluminosilicate material with a comparable strength performance to the Ordinary Portland Cement, and are considered to be a good potential substitute for the latter [8]. The low permeability, resistance to acid attack and long-term durability of geopolymers make it an excellent potential in solidification/stabilization of the hazardous wastes [9,10]. In recent years, the immobilization of organic wastes in geopolymer materials has been extensively studied [11]. Cantarel et al. [12] studied the influence of liquid oil waste on the mechanical performance and leaching rate of organic materials. The results showed that the cumulative quantity of oil compounds released in the leachate at 30 days does not exceed 0.19% of the organic material initially encapsulated in the sample. Al-Mashaqbeh et al. [13] investigated the immobilization of organic dyes in geopolymer. It was found that all kinds of dyes can been immobilized in geopolymers, and the efficiency is higher than 94%. Therefore, it is feasible to use geopolymer technology to immobilize the high content of organic contaminants in spent caustic. In addition, geopolymers are synthesized through activating the raw materials with a highly alkaline solution [14]. In the life cycle assessment of geopolymers, the use of alkali activators contributed the highest CO_2_ footprint in most life cycle categories and was also associated with high costs [15,16]. Daniel et al. also elaborated the life cycle assessment of a geopolymer concrete, and they reported that the use of alkali activators represents the most important environmental burden [17]. Fortunately, the mass fraction of NaOH and Na_2_CO_3_ in the treated spent caustic can reach about 20% [18], which has the potential to partially replace the alkali activator to prepare geopolymers, and can further reduce the cost and the CO_2_ emission of geopolymer preparation.

In this work, we have studied the preparation of geopolymers using spent caustic to partially replace NaOH solution as an alkali activator, and pure NaOH solution with comparable alkalinity was used as a control group. The effect of spent caustic on the compressive strength and microstructure of geopolymers was studied, and the immobilization efficiency of organics in geopolymers was evaluated. This study proposes a new method for the utilization of spent caustic, which also reduces the preparation cost of geopolymers.

## 2. Experimental

### 2.1. Materials

**Figure 1:**
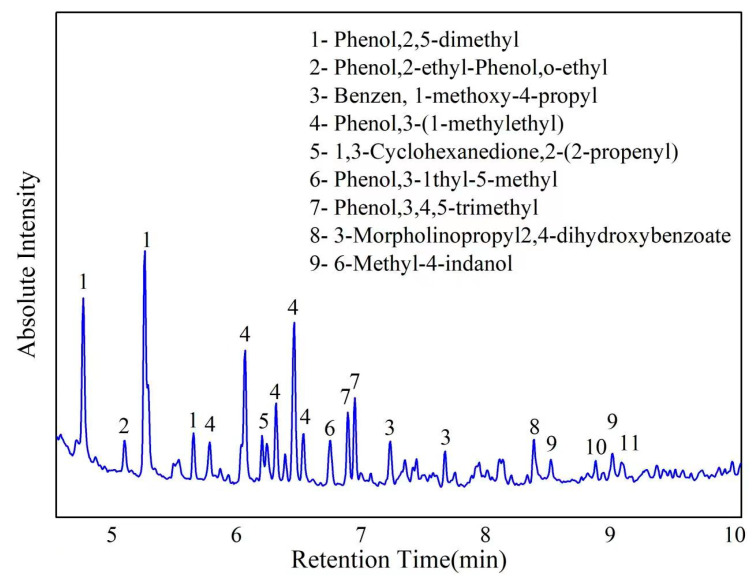
GC analysis of spent caustic.

The class F fly ash used in this paper was purchased from a plant in Hubei province, China, which was used as a raw material in the synthesis of geopolymers. Table 1 shows the chemical analysis of fly ash measured by XRF. The spent caustic was provided from Sinopec Wuhan petrochemical company. The organics in spent caustic measured by gas chromatography spectrometry (GC, LEEMAN LABS Clarus 680) are shown in Figure 1, in which a large amount of organic matter appears. The concentration of organic total organic carbon (TOC) in spent caustic is 4312 ppm, indicating large quantities of organics in it. The alkalinity of spent caustic is equivalent to 2.08 mol/L NaOH solution, which was evaluated by titrating with HCl solution until pH = 7. This can be attributed to the incomplete oxidation of organics during the WAO process [19]. The sodium hydroxide (ACS reagent grade) was used as alkali activator.

### 2.2. Synthesis of Geopolymers

**Figure 2:**
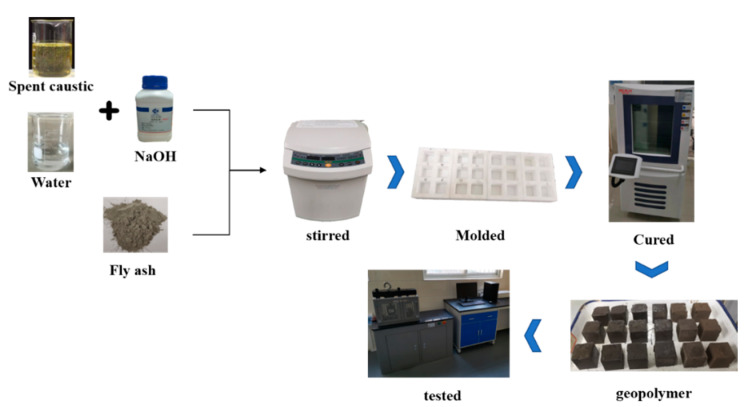
The diagram of the geopolymer preparation process.

The activator solution was prepared by mixing NaOH with spent caustic or deionized water in different proportions. Figure 2 shows the diagram of the geopolymer preparation process. In a typical synthesis of geopolymers, the fly ash and alkali activator was stirred in the homogeneous mixture and poured into a 5 cm × 5 cm × 5 cm steel mold. After being vibrated on a vibration table to release the air bubble, the mold was sealed with polyethylene film and cured at 60 °C for 6 h. Then, the samples were demolded and sealed in plastic bags in the natural environment for 7 days. The diagram of the geopolymer preparation process is shown in Table 2. In the preparation of geopolymers with spent caustic, the amount of fly ash and spent caustic was constant, at 100 g and 25 mL, respectively; the content of NaOH was the unique variable from 0 g to 12 g. A series of samples synthesized with the same alkalinity prepared with NaOH were used for comparison.

### 2.3. Immobilization of Organic Contamination

According to the Chinese Ministry of Environmental Protection Standard HJ/T 300–2007, the immobilization efficiency of organics in geopolymers was evaluated by the leaching toxicity of organics. In this method, the leaching process was performed with the liquid/solid ratio of 20 mL/g for 18 h, which is the same as it in the USEPA TCLP method. However, the extraction solution was prepared by dissolving 17.25 mL acetic acid into 1 L deionized water, and the amount of acid was almost three times that of the latter, so this standard is more severe. In addition, a zero-headspace extraction vessel was used to prevent the volatilization during the leaching process; the specific operation refers to Reference [20]. The leachate was filtered through a 0.45 μm filter. The amount of organic contamination in the extraction liquid, spent caustic and leachate were analyzed by measuring the total organic carbon (TOC).

### 2.4. Characterization of Geopolymers

The mechanical property of geopolymers was tested by a mechanical tester (Hangzhou Xingo Technology, EHC-1300) in accordance with the ASTM Test C-39. In the measurement, at least three specimens were tested, and the average value was used. The mineralogical study of geopolymers was characterized by X-ray diffraction (XRD, Brucker D8) with Cu Ka radiation. The pore distribution of geopolymers was analyzed by mercury intrusion porosimetry analysis (MIP, AutoPore Ⅳ 9520). ^29^Si nuclear magnetic resonance (NMR, Bruker AVANCE III) spectra were used to analyze the microstructure of geopolymer gel. The spectra were collected at 79.5 kHz on a 7 mm probe with a spinning speed of 5 kHz, a pulse width of 6.5 μs and a relaxation delay of 10 s. The ^29^Si chemical shift was referenced to an external standard of tetramethylsilane.

## 3. Results and Discussion

### 3.1. Compressive Strength

**Figure 3:**
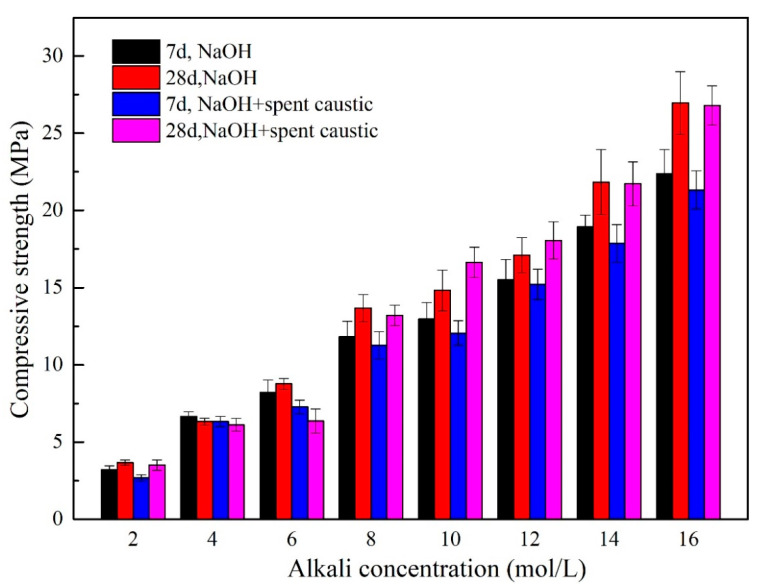
The compressive strength of geopolymers synthesized with different alkali activators.

Figure 3 shows the compressive strength of geopolymers synthesized with different alkali activators. The compressive strength of geopolymers activated with only spent caustic is 2.67 MPa in 7 d, which indicates that spent caustic can be used as an alkali activator for geopolymerization. With the alkalinity of alkali solution increasing from 2 mol/L to 14 mol/L, the compressive strength of geopolymers increased to 21.86 MPa. The 7-day compressive strength of all samples with spent caustic are less than that of the samples without spent caustic at the same degree of alkalinity. This may be due to the fact that the organics in the spent caustic hinder the hydration of geopolymers and reduce the strength of the product [12]. For compressive strength at 28 days, the geopolymer with spent caustic is also lower than that without spent caustic when the alkalinity is 2 mol/L. However, with the degree of alkalinity increasing from 4 mol/L to 10 mol/L, the compressive strength significantly increased, and the geopolymer with spent caustic is higher than that without spent caustic. Considering that the main component in spent caustic is NaOH and Na_2_CO_3_, it can be attributed to the combined effect of Na_2_CO_3_ and NaOH as alkaline activator in preparation of geopolymers. Zhang et al. [21] investigated different alkali-activators on the performance of alkali-activated nickel slag, and they also reported that the NaOH/Na_2_CO_3_-activated system showed higher compressive and flexural strengths than the NaOH-activated system. When the alkalinity is further increased to higher than 12 mol/L, the proportion of alkali in the spent caustic is much less than that of sodium hydroxide, which has less of an effect on the geopolymer, resulting in a similar strength.

### 3.2. X-ray Diffraction (XRD)

**Figure 4:**
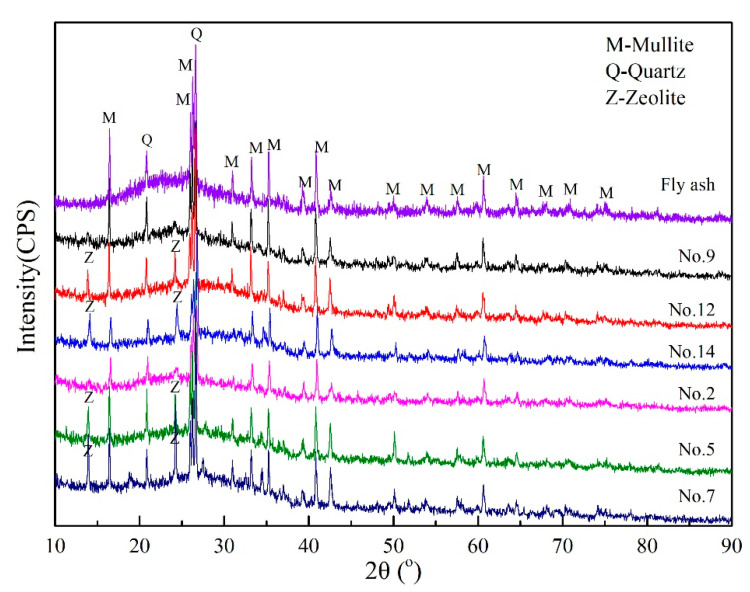
The X-ray diffractograms of fly ash and geopolymers synthesized with different alkali activators.

In order to study the activation effect of spent caustic on the structure of fly ash-based geopolymers, XRD analyses carried out on NaOH-activated geopolymers and spent caustic-activated geopolymers is shown in Figure 4. In fly ash, there is a typical dispersion peak of amorphous aluminosilicate at the position of 20–25° with crystal structures of mullite (Reference code: 01-079-1457) and quartz (Reference code: 01-070-3755). For geopolymers, the intensity of the mullite peak in the raw material is weakened, and the dispersion peak in fly ash shifted to a high angle at the position of 27–29°, which is a characteristic peak of geopolymer gel. The diffraction spectra of the geopolymers activated by spent caustic and pure NaOH solution showed the similar characteristic peak, indicating that spent caustic can partially replace NaOH as the alkali activator under the same degree of alkalinity, and the impurities such as organic in the spent caustic have little effect on the structure of the geopolymer. In addition, with the increase in the alkali activator concentration, the characteristic peak of zeolite appeared in the sample and the intensity gradually increased. It is consistent with previous research results that zeolites formation is as a result of the reaction parallel to geopolymerization [22].

### 3.3. Nuclear Magnetic Resolution (NMR) Spectra

**Figure 5:**
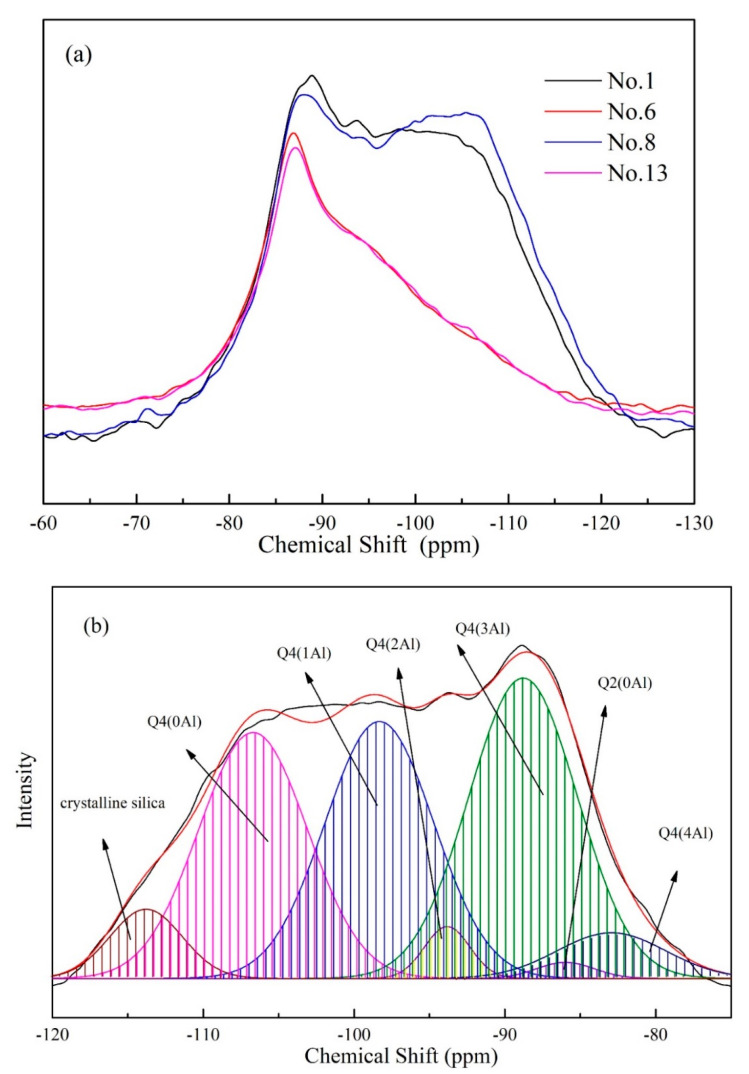

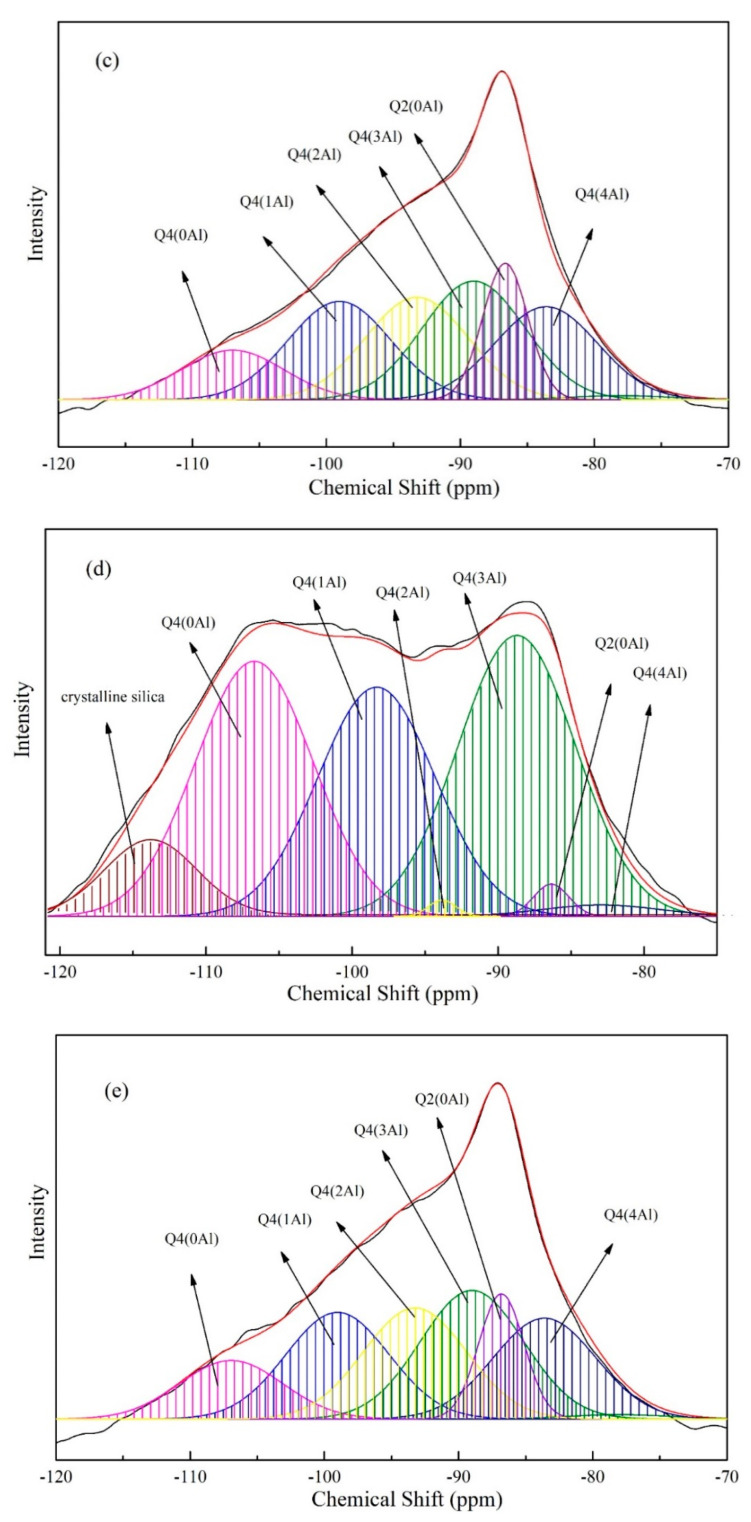
^29^Si NMR spectra (**a**) and their deconvolution of geopolymers with synthesized with different alkali activators, No. 1 (**b**), No. 6 (**c**), No. 8 (**d**) and No. 13 (**e**).

Figure 5 showed the ^29^Si NMR spectra of geopolymers synthesized with different alkali activator after 28 days. The Gaussian peak deconvolution of this spectrum can be used to separate and quantify Q^4^(mAl) species of silicon (0 ≤ m ≤ n ≤ 4, m) [23]. The resonance of a Q^4^(mAl) center with the replacement of each aluminum by silicon is an approximate −5 ppm difference in δ, with Q^4^(4Al), Q^4^(3Al), Q^4^(2Al), Q^4^(1Al) and Q^4^(0Al) resonating at approximately −84, −89, −93, −99 and −108 ppm, respectively [24]. It was also observed that the peak of −86.8 ppm was related to the Q^2^ structure in C-S-H gel [25]. The peaks beyond −110 ppm represent the different crystalline silica phases in fly ash [26]. Table 3 presents ^29^Si NMR date for the samples after deconvolution. For the samples No. 3 and No. 10, a broad peak ranged from −70 to −120 ppm was shown, which is similar to the spectrum of fly ash. It indicated that a large number of fly ash was not activated to form geopolymer gel. Compared with sample No. 10, the content of Q^4^(4Al) and Q^4^(3Al) in sample No. 3 is higher, while that of Q^4^(0Al) and Crystalline silica is opposite. Considering that the Al-rich gel is the primary reaction product of the alkali activation of fly ash [27], it suggested that the addition of spent caustic promotes the decomposition of fly ash and the formation of geopolymers at a low degree of alkalinity, which is consistent with the results of compressive strength. For the samples No. 8 and No. 13, the structure of the geopolymer gel was similar, and it indicated that the effect of the alkali activator with spent caustic is the same as that with pure NaOH solution, and the organic in spent caustic has no influence on the long-term development of geopolymers.

### 3.4. Pore Structure

**Figure 6:**
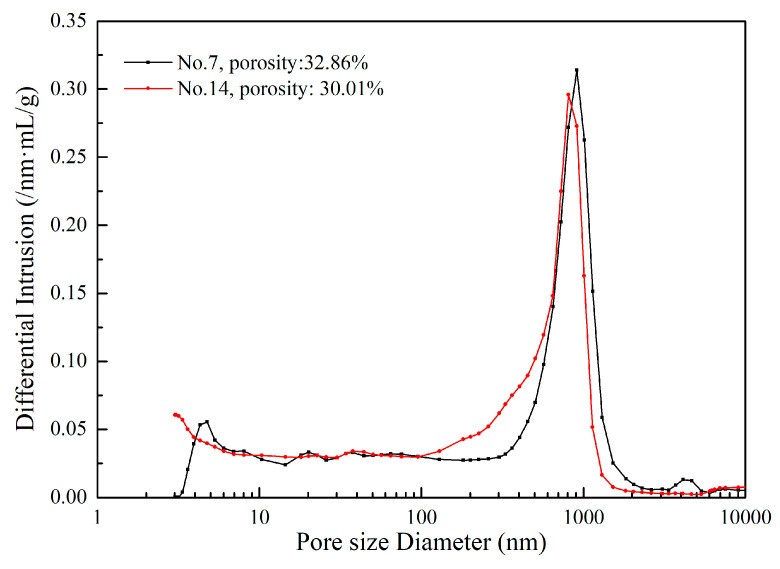
The porosity and pore size distribution of geopolymers synthesized with different alkali activator.

The porosity and pore distribution of geopolymers with different alkali activators is shown in Figure 6. Under the same alkalinity condition, the porosity and pore size distribution of different geopolymers are similar, indicating that the alkali in the spent caustic has the same activation effect to NaOH. This resulted in a similar structure of the formed geopolymers, which is consistent with the results of XRD and NMR. However, the pore with a size between 100–500 nm decreased, while the number of pores with a size above 1 um increased. It leads to a slight increase in the porosity from 30.01% to 32.86%, but this has no noticeable effect on compressive strength.

### 3.5. Organics Immobilization

**Figure 7:**
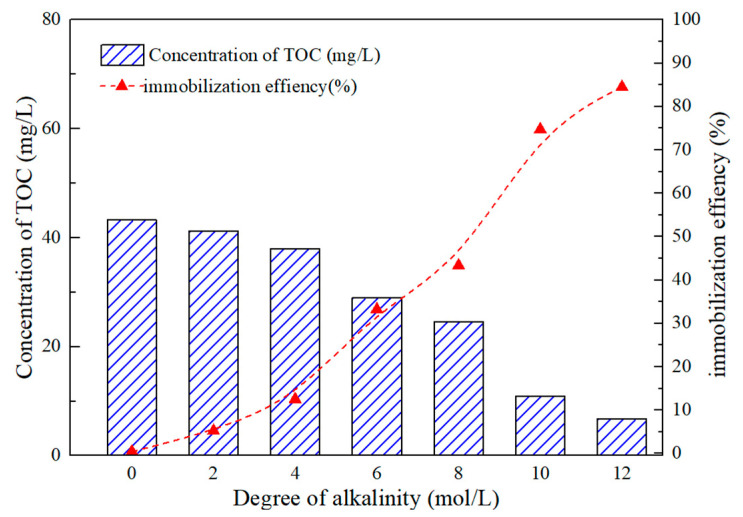
The concentration of TOC in the leachate and the organics immobilization efficiency of geopolymers synthesized with different alkali activator.

The measurement of total organic carbon (TOC) was used to quantify the leaching of organics from geopolymers [28]. The organics immobilization efficiency of geopolymers was given in Figure 7. As the content of NaOH increased from 2 mol to 14 mol, the concentration of organics in the leachate decreased from 43.24 to 6.72 mg/L, and the immobilization efficiency increased from 0.4% to 84.5%. It suggests that the geopolymers can effectively immobilize the organics in the spent caustic, and the immobilization efficiency shows positive correlation with degree of alkalinity. This may be due to the formation of more zeolite structures in the process of activating fly ash with high concentration of alkali activator, and the adsorption of zeolite is the main way for geopolymers to immobilize organics [29]. In a previous study, Al-Mashaqbeh et al. [13] reported that geopolymers can be used to immobilized organic dyes, and the immobilization efficiency is 94%, which is higher than found in this work. Considering that the concentration of organic dye is 3000 mg/L, which is much lower than the 4341 mg/L level in this study, this result is acceptable. In addition, the Si/Al ratio has a significant impact on the immobilization of organics, and in further study, we will investigate its effect on the immobilization of organics in spent caustic.

## 4. Conclusions

In this study, fly ash-based geopolymers were prepared by using spent caustic as alkali activator and the influence of spent caustic on the formation of geopolymers was studied. The result is as follows:

(1) The spent caustic can partially replace the NaOH to synthesize geopolymers, and the organics in spent caustic can be immobilized in geopolymers. The method can not only make the spent caustic harmless, but also utilize the strong alkalinity of the spent caustic to reduce the cost of preparing the geopolymer.
(2) When the degree of alkalinity is higher than 4 mol/L, the geopolymers prepared by the mixed activator of spent caustic and sodium hydroxide have better 28 days compressive strength than that synthesized with pure NaOH solution, and the highest strength can reach 21.86 Mpa.
(3) With the degree of alkalinity increasing, the immobilization efficiency of organics in geopolymers is improved, and the maximum can reach 84.5%. The organics in the spent caustic will hinder geopolymerization at the initial stage but has little effect on the chemical structure and phase of the final product.

## Figures and Tables

**Table 1:** Chemical composition of fly ash.

Component	SiO_2_	Al_2_O_3_	K_2_O	Fe_2_O_3_	TiO_2_	CaO	MgO	LOI
wt%	49.18	33.80	2.28	4.89	0.73	4.84	0.81	3.47

**Table 2:** Preparation regime of geopolymers.

Specimen No.	Fly Ash	NaOH	Spent Caustic	Water
1	100 g	0 g	25 mL	/
2		2 g		
3		4 g		
4		6 g		
5		8 g		
6		10 g		
7		12 g		
8		2 g	/	25 mL
9		4 g		
10		6 g		
11		8 g		
12		10 g		
13		12 g		
14		14 g		

**Table 3:** Deconvolution results of ^29^Si MAS NMR spectra of different geopolymers.

Sample	No. 3	No. 8	No. 10	No. 13
Q^4^(0Al)	26.96%	1.42%	30.07%	10.44%
Q^4^(1Al)	28.18%	2.59%	27.01%	19.02%
Q^4^(2Al)	3.14%	26.96%	0.45%	19.81%
Q^4^(3Al)	30.73%	31.20%	33.13%	22.93%
Q^4^(4Al)	5.66%	24.47	1.35%	17.98%
Q^2^(0Al)	1.03%	13.36%	1.14%	9.82%
Crystalline silica	4.30%	/	6.85%	/

## Data Availability

The data presented in this study are available from the corresponding author upon request.
